# Comparable Stocks, Boundedly Rational Stock Markets and IPO Entry Rates

**DOI:** 10.1371/journal.pone.0061474

**Published:** 2013-05-17

**Authors:** Jay Chok, Jifeng Qian

**Affiliations:** Keck Graduate Institute, Claremont, California, United States of America; University of Warwick, United Kingdom

## Abstract

In this study, we examine how initial public offerings (IPO) entry rates are affected when stock markets are boundedly rational and IPO firms infer information from their counterparts in the market. We hypothesize a curvilinear relationship between the number of comparable stocks and initial public offerings (IPO) entry rates into the NASDAQ Stock Exchange. Furthermore, we argue that trading volume and changes in stock returns partially mediates the relationship between the number of comparable stocks and IPO entry rates. The statistical evidence provides strong support for the hypotheses.

## Introduction

This paper integrates two sociologists' perspectives. One perspective is that stock markets are boundedly rational [1]. The other perspective is that actors infer information from their counterparts in the market [2]. Based on these two perspectives, we hypothesized a curvilinear relationship between the number of comparable stocks and initial public offerings (i.e. IPO) entry rates in a particular market tier within the NASDAQ Stock Exchange (see Note 1 in Footnotes S1). If the stock market is boundedly rational, it doesn't matter whether the stock change is positive or negative. Finally, we argued that trading volume and changes in stock returns partially mediates the relationship between the number of comparable stocks and IPO entry rate.

Prior literature in the economics of information generally rests on the foundation that when buyers and sellers are interacting for the first time in the presence of information asymmetry (i.e. the seller knows more than the buyer), information asymmetry disadvantages the seller because buyers account for the probability that the product sold by the seller is of dubious quality [3]. The buyer thus adjusts the price s/he is willing to pay accordingly. The price adjustment drives out the high quality goods progressively and buyers adjust the prices continually as the quality falls. In the extreme, this results in market collapse.

This stream of “adverse selection” research had been applied to the IPO research in finance [4]. In general, firms going public for the first time face a “lemons” problem wherein investors know less about the firm going public than the insiders (i.e. managers) who are helping the firm to go public (i.e. sell shares to the public for the first time). Accordingly, there is greater information asymmetry in the primary capital markets than the secondary capital markets, which adversely affect initial stock pricing. However, key mechanisms related to information asymmetry had focused on under-pricing [5] and incidentally, there is very little research in IPO entry rates at the population level (see Note 2 in Footnotes S1). Fundamentally, such a view focuses on the interaction between a firm and its potential investors.

In contrast, White [2] developed a sociological view of markets wherein economists' neoclassical theory of the firm is embedded within a sociological view of markets. In this view, markets are self-reproducing social structures among specific cliques of firms who evolve roles from observations of each other's behavior. White [2] focuses on production and argued that firms' production decisions is based on the observed positions of all other producers rather than that of consumers. The logic implies that it is possible that non-public firms' decisions to enter the public equities market are dependent on the availability of information about observed positions of the public firms in their reference groups. Accordingly, a market sector is more likely to experience entry when there is sufficient information for the non-public firms to make such decisions.

In fact, as long as there is asset correlation among private and public firms of the same industry, it is possible to infer information about a privately held firm by observing its publicly held counterpart. Indeed, recent empirical evidence suggests that there are information-driven co-movement of stock prices in the stock markets [6]. Thus, it follows that firms should be motivated to go public when the number of comparable stocks in their sector is high. Moreover, it is not necessary that the stock change is positive. If the stock market is rational, valuable information can be inferred from both positive and negative events (see Note 3 in Footnotes S1). Therefore, we hypothesized that IPO entry rate is positively associated with recent stock changes; both positive and negative. However, when the stock market is boundedly rational, the benefit to having a larger number of comparable stocks is limited. Stock markets are boundedly rational when they are not efficient. According to the efficient market hypotheses (EMH), there are three forms of market efficiency. In weak-form efficiency, future prices cannot be predicted on the basis of historical prices. In semi-strong efficiency, neither fundamental analyses nor technical analyses can produce returns in excess of the expected rate of returns because financial asset prices adjust rapidly to publicly available information in an unbiased fashion. In strong-form efficiency, the prices reflect all public and private information. EMH assume that traders have rational expectations, which means that they are able to incorporate information about future expectations into asset prices. It is assumed that traders do not make systematic errors when predicting the future. Individual traders are allowed to have inaccurate expectations but these erroneous expectations are random. Furthermore, when new information appears, traders update their expectations appropriately. This means that on average, the population of traders prices the financial assets correctly even though individual traders may deviate from perfect foresight in a random manner.

Hommes and colleagues [7] suggest that the EMH and rational expectations do not describe empirical reality. Instead, future expectations about asset prices are shaped by each trader's guestimates about other traders' expectations. In particular, they were concerned with how economic agents rely on the firm's historical prices to predict its future prices (as indicated in equation 7, page 11 in their manuscript). This type of trading strategy is an instance of bounded rationality. They conduct simulation studies and show that trading strategies such as fundamental and technical analyses can earn abnormal returns. Their results suggest that individual traders are boundedly rational, meaning that they could use trading strategies that rely on historical information that result in systematic errors, are not driven out of the market.

In essence, a market can be viewed as an expectations feedback system: past market behavior determines individual expectations which, in turn, determine current market behavior and so on. Individual's current expected price can be estimated by averaged previous prices and its own previous observed price.

By way of context, our work differs from Hommes and colleagues in that an IPO firm does not have a history of publicly-traded stock prices and led us to offer a different explanation. When there is a larger sample of comparable stocks, pricing error is minimized and therefore, the IPO firm is more likely to go public. That is, in the absence of the firm's own historical prices, it makes pricing-related inferences based on its' publicly traded competitors' stock prices. This specific reliance on public information about peers differs from the scholarly work of Hommes and colleagues and fills a gap in the literature.

Our explanation is related to recent research on the economic sociology of the financial markets. For example, Zajac and Westphal [1] document that market reactions in financial markets, to a certain extent, are socially constructed. They studied the Fortune 500 companies, which are so large that the market may have excess information on them. Although the market is, to a certain extent, capable of processing information and inputting that information into the stock prices, the market is boundedly rational. Thus, market reactions to shares repurchase plans are affected by competing ideologies that either promote or discourage shares repurchase plans. These ideologies are also known as institutional logics – belief systems that shape behavior [8]. The institutional logic in an earlier era was that managers can skillfully allocate internal capital to generate superior returns whereas in the current era, another institutional logic, also known as the shareholder logic, dominates [9]. The shareholder logic is a belief system buttressed by the agency theory developed by Jensen and Meckling [10]. In particular, proponents of the shareholder logic argue that the interests of managers are not fully aligned with that of investors. It is better for the managers to disgorge excess free cash flows, which can be more efficiently allocated by investors in the financial markets. They also argued that firms that engage in institutional decoupling by announcing repurchase plans but did not implement the announced intent were nonetheless able to experience increases in market value when an increasing number of firms behave in this manner. This suggests that the stock markets are not able to absorb all information.

Related studies [11], [12] suggest that social identities for firms are important. Rao, Davis and Ward [11] argued that organizations signal their social identity through their affiliations with stock exchanges. Their qualitative research suggests that investor-relations managers see social identities as important for presenting the public corporation's image to the investors. Moreover, to the extent that public corporations with social identities similar to the focal firm defect to rival stock exchanges, recursive information is generated. Investors infer information about the firms that defected and stock prices of defected firms in turn affect the firms that did not defect. Again, Rao, Davis and Ward [11]'s focus is on firms that are sufficiently large that it may be appropriate to migrate from NASDAQ to NYSE.

Similarly, Zuckerman [12] suggests that public corporations need to have coherent identities in the stock markets. He document that firms not covered by securities analysts that specialize in its industries suffered penalties with regard to stock prices. That is, the lack of comparable stock on a stock by specialized securities analysts reflects confusion over the stock's identity with regard to standard industry classification and that the illegitimacy associated with the identity confusion should depress demand for the stock. He suggests that there is significant uncertainty in the capital markets and investors rely on mediated information providers like securities analysts, investor-relations managers and corporate executives to make sense of the capital markets. This suggests a view of a boundedly rational stock market.

Work by Zuckerman [13] point to the same direction. Zuckerman [13] argued that comparison between a stock's position in the network of coverage by securities analysts and its industry classification code revealed whether the stock is coherently classified. This affects the efficiency of the price-setting process for each stock. Indeed, Zuckerman document that both volume and volatility, proxies for information in financial economics, are higher for incoherent stocks [13], [14].

It is useful to note that information produced by securities analysts is not necessarily more accurate than that produced via transactions among traders. The key difference is of a symbolic nature; it increases coherence and economizes on information processing. For example, Rao, Greve and Davis [15] document that securities analysts' decisions to initiate and abandon coverage of firms listed on the NASDAQ national market are influenced by their peers' decisions to provide coverage. Securities analysts frequently initiate coverage of a firm because their peers are doing so and are particularly prone to overestimating the covered firm's future profitability. Due to misjudgment, these analysts are subsequently more likely to abandon coverage of the firm.

To recap, the analyses suggest that while availability of comparable stocks facilitates IPO entry, information overload may discourage entry if the stock market is boundedly rational. Thus, we hypothesize that there is a curvilinear relationship between recent and IPO entry rate. That is, increasing comparable stock facilitates entry but after a certain point, too much information discourages entry.

Finally, Haveman [16] demonstrate that density and entry rates have a curvilinear relationship. The general argument of this density-dependence model of competition and legitimation is that increasing numbers of firms in a field encourages entry that would otherwise be inhibited by the liability of newness because the increasing density provides cognitive legitimacy [17]. However, beyond a certain point, competition sets in and entry rates fall as prospective firms increasingly find it difficult to survive in a highly competitive environment. We argued that part of the reason that density has a curvilinear relationship with entry is that density also generates information. As mentioned earlier, increasing the number of comparable stock in the field encourages entry but having too many peer stocks to compare discourages entry due to information overload. View from this angle, density has an additional impact on IPO entry beyond legitimation and competition. Accordingly, we hypothesize that trading volume and changes in stock returns partially mediates the relationship between the number of comparable stocks and IPO entry rate. To summarize, while previous work dealt with how economic agents rely on the firm's historical prices to predict its future prices, we focus on the idea that in the absence of the firm's own historical prices, it is possible to make pricing-related inferences based on a firm's publicly traded competitors' stock prices. This specific reliance on public information about peers differs from the work of Hommes and colleagues and thus fills a gap in the literature.

## Materials and Methods

Data on the variables is downloaded from the SDC Platinum database and CRSP (i.e. Center for Research in Security Prices).

Table 1 indicates the variables that we created from the raw dataset. This is a panel dataset that is summarized to the 2-digit SIC group level and monthly time series period (i.e. April 1982 to June 2006). The data available allows us to study this market tier from birth to closure. That is, there will not be left-truncation.

**Table 1:** Distribution of observations before collapsing the dataset.

Listing Status	Frequencies	Percent	Cumulative
active	668,795	89.41	89.41
only one market maker	68	0.01	89.41
suspended	848	0.11	89.53
inactive	455	0.06	89.59
de-listed	77,879	10.41	100
Total	748,045	100	

The raw sample in CRSP consists only of common stock securities listed on the NASDAQ National Market System. Monthly data on stock returns, trading volume, 2-digit SIC code and time period are downloaded. Information on the listing status of the common stocks at any given month could be one of these six categories: unknown, active, only one market maker, suspended, inactive and de-listed. In the month that any given stock is de-listed or suspended, stock returns and trading volume would still be available. After that, de-listed stocks would no longer be tracked. No common stock in our sample is labeled with unknown status. As shown in table 1 below, most of the observations are labeled as active.

The raw data from SDC Platinum consists of IPO deals within United States. SDC Platinum defined IPO relatively broadly to include deals that are not technically initial public offerings. That is, a firm may already had a primary listing before in a particular stock exchange but are listed for the first time in another stock exchange (a table showing the number of IPOs we observed over time is provided in table S1). This dataset has firm-level observations with issue dates and a series of dummy variables indicating whether the deals are initial public offerings, common stock offerings, and foreign issues. We also have information on the number of primary and secondary shares offered. The deals include listings on the NASDAQ National Market System (NMS) and the Small Capital Market (SCM). Using these variables, we construct a dependent variable that is defined as the presence of primary common stock initial public offering in the NASDAQ National Market System for each 2-digit SIC group in a given time period. Table 2 provides definitions of the basic notations that go into constructing the variables' notations.

**Table 2:** Basic notation definition.

Name /Notation	Definition
NMS	National Market System
i	Permno observation
j	The two-digit SIC group
t	The time period (month and year)
year	The time period (year)
p	The probability of a primary common stock initial public offering in the NASDAQ NMS
ret	Monthly stock returns per stock in the NASDAQ NMS
vol	Monthly trading volume per stock in the NASDAQ NMS
n	Number of NASDAQ NMS common stocks
*k_jt_*	Dummy variable coded as 1 if the particular 2-digit SIC group experiences at least one primary common stock initial public offering in the NASDAQ NMS and 0 if otherwise.
IPO_i_	An initial public offering

Table 3 provides the label and notations of the basic variables that go into the panel logistic regressions. IPO entry is defined as the probability of a primary common stock initial public offering in the NASDAQ NMS per SIC group within a given time period. Number of IPOs is the number of primary common stock initial public offering in the NASDAQ NMS for each 2-digit SIC group in a given time period. ARET is the average monthly stock return for each 2-digit SIC group in a given time period. SDRET is the cross-sectional standard deviation of the individual stock returns for each 2-digit SIC group in a given time period. AVOL is the average monthly trading volume for a particular two-digit SIC group normalized by the largest trading volume in that particular year. SDVOL is the cross-sectional standard deviation of the monthly trading volume for all stocks within the particular two-digit SIC group normalized by the largest standard deviation in that particular year. Positive is the number of positive changes within the particular two-digit SIC group in a given time period where x is coded as 1 if the stock returns of a particular stock is positive and 0 if otherwise. Negative is the Number of negative changes within the particular two-digit SIC group in a given time period where y is coded as 1 if the stock returns of a particular stock is negative and 0 if otherwise. Density is the number of NASDAQ NMS common stock for each 2-digit SIC group in a given time period.

**Table 3:** Variable label, notation and definition.

Variable Label	Notation
IPO entry	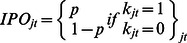
Number of IPOs	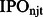
Average monthly stock return	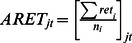
Cross-sectional standard deviation of stock returns	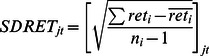
Average monthly trading volume	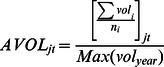
Cross-sectional standard deviation of trading volume	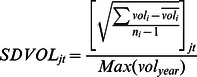
Number of positive changes	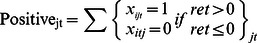
Number of negative changes	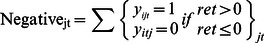
Number of common stocks	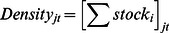

**Figure 1:**
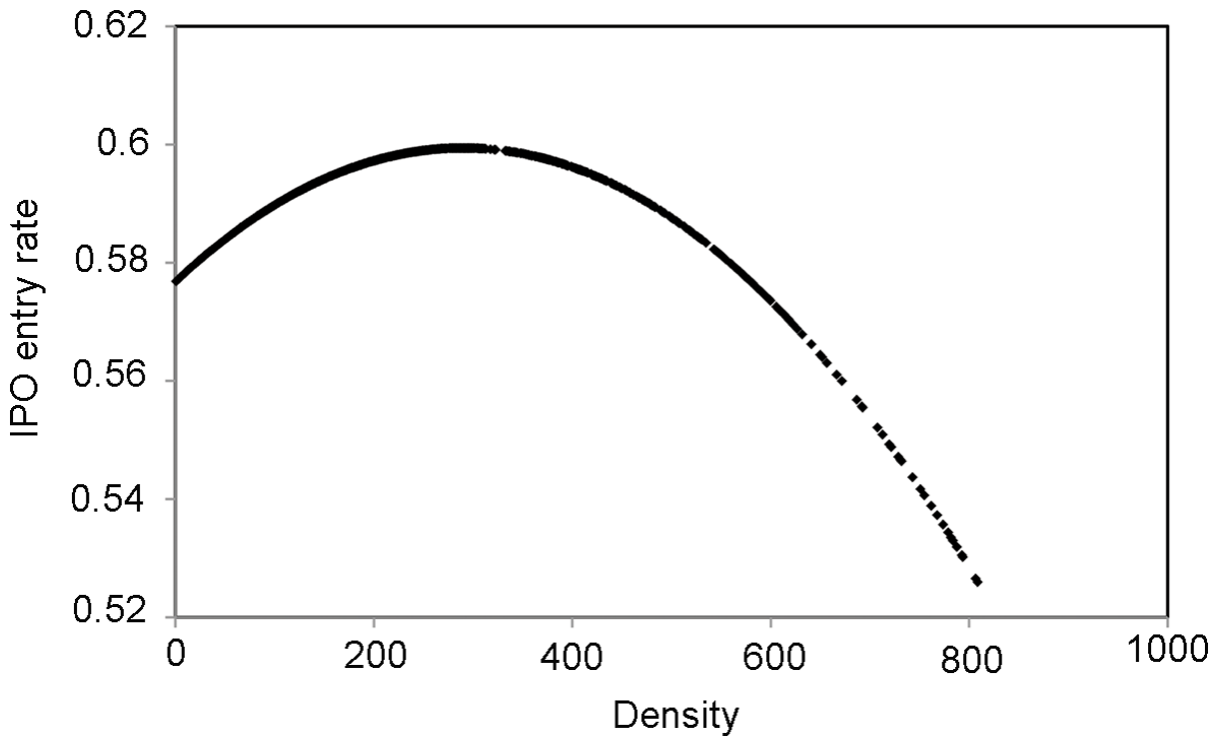
IPO entry rate vs. Density. There is a curvilinear relationship between the number of comparable stocks (density) and initial public offerings (IPO) entry rates into the NASDAQ Stock Exchange.

To construct the simplified logistic model, we use density (number of comparable stock) and density squared in the previous time span (mostly previous month) as independent variables. We use IPO entry as the dependent variable (figure 1). We then construct a more sophisticated regression model. Independent variables that proxy for information are: average trading volume in a 2-digit SIC sector, cross-sectional standard deviation of trading volume in a 2-digit SIC sector, number of stocks with positive stock returns in a 2-digit SIC sector and number of stocks with negative stock returns in a 2-digit SIC sector. Because we propose a curvilinear effect, we also square the terms.

Trading volume is a standard proxy for information arrival, heterogeneous belief among investors and liquidity in stock markets [14], [18], [19]. In this paper, we use trading volume as a proxy for information. In order not to confound with size effects, we took the average trading volume. In addition, recent research also suggests that the cross-sectional variation of trading volume contains valuable information about stock-specific characteristics such as risk, size, price, trading costs and membership in stock indexes [18]. To the extent that trading volumes among stocks within a 2-digit SIC sector in the cross-section are sufficiently different, there will be more unique information about stocks in that sector. In contrast, in a 2-digit SIC sector where the trading volumes among stocks in the cross-section are sufficiently similar, there should be more redundant information. Hence, we also calculate the cross-sectional standard deviation of trading volume for all stocks within each 2-digit SIC group.

Stock prices and hence stock returns are also traditionally assumed to contain information [20]. In order to show that positive and negative information are both informative for IPO entry, we create variables that are, within a given 2-digit SIC sector, the number of stocks with positive and negative stock returns respectively.

The control variables are average stock returns in a 2-digit SIC sector, cross-sectional standard deviation in the stock returns of each 2-digit SIC sector, the number of IPO entries in the prior period, density and density squared. These measures represent the market conditions facing each 2-digit SIC sector for each month. In particular, density and density squared proxy for the competition and cognitive legitimacy affecting each 2-digit SIC sector every month. The number of IPO entries in the prior period represents a strong control for the probability that a 2-digit SIC sector will experienced IPO entry in the current period. Finally, cross-sectional standard deviation in the stock returns proxy for the identity coherence of each 2-digit SIC sector. Sectors where common stocks' returns rise and fall in together would have much simpler identities and thus greater identity coherence.

The probability of a sector experiencing an IPO entry is modeled as a function of a vector of control variables (C) and a vector of hypothesized variables (I):
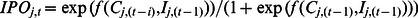
(1)


The regression equation is:
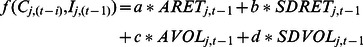


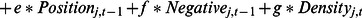
(2)


In this equation, a, b, c, d, e, f, and g are constants. Refer to table 3 for definition of other terms.

## Results and Discussion

Compared to using only density (i.e. the number of comparable stocks) as a predictor, the logistic model that takes into account both density and density squared performs significantly better. This suggests, as indicated in figure 1 above, that there is a curvilinear relationship between density and IPO entry.

Table 4 shows the partial correlations of the basic independent variables (see Note 4 in Footnotes S1). This is a simplified view as the reported correlation between two variables in this table only holds constant the effects of other variables in this table. The square terms of the variables are not controlled for. It also does not take into consideration sector and period effects. Most of the partial correlations are highly statistical significant, which provides some confidence with regard to the accuracy of the correlations. Most of the partial correlations among the variables are not high. However, at the 2-digit SIC level, average monthly trading volume is highly correlated (0.839) with the cross-sectional standard deviation in monthly trading volume among individual stocks. In addition, the correlation between the number of positive changes and the number of negative changes is also high (0.616). Hence, in our econometric equations, we do not use both measures of trading volume together and likewise for the number of positive/negative changes.

**Table 4:** Partial Correlations.

	Number of IPOs	Number of common stocks	Average monthly stock return	Cross-sectional standard deviation of stock returns	Average monthly trading volume	Cross-sectional standard deviation of trading volume	Number of positive changes
Number of common stocks	0.246^1^						
Average monthly stock return	0.010	−0.057^1^					
Cross-sectional standard deviation of stock returns	0.014^4^	0.120^1^	0.259^1^				
Average monthly trading volume	0.106^1^	−0.052^1^	0.022^2^	0.016^3^			
Cross-sectional standard deviation of trading volume	0.074^1^	0.066^1^	−0.019^3^	0.073^1^	0.839^1^		
Number of positive changes	0.000	0.360^1^	0.223^1^	−0.069^1^	−0.022^3^	0.006	
Number of negative changes	0.033^1^	0.306^1^	−0.189^1^	0.025^2^	0.043^1^	0.130^1^	0.616^1^

1 significant at 0.1%,^2^ significant at 1%,^3^ significant at 5%;^4^ significant at 10%.

In table 5, we run preliminary mediation tests to tease out the effects that trading volume and changes in stock returns have on the number of comparable stocks and subsequently on IPO entry rates. In each of the mediation tests, the presence of primary common stock IPOs in the NASDAQ NMS for each 2-digit SIC group in a given time period is set up as the dependent variable. We estimate mediation tests for comparable stocks by entering several variables separately as mediators. These variables are average trading volume, cross-sectional standard deviation of trading volume, number of positive changes, and number of negative changes. The mediation analyses test the relationship holding constant IPO entries in the prior period, the number of common stocks squared, average monthly stock returns and the cross-sectional standard deviation in the stock returns. The results are highly statistically significant. In particular, changes (positive or negative) accounts for around 15 to 30% of the effects that is mediated while trading volume accounts for 3 to 4% of the effects that is mediated. We repeat the analyses with Goodman mediation tests, which yield very similar results.

**Table 5:** Sobel Mediation Tests for the information proxy variables.

	Z-statistic	P values	Percent of total effect that is mediated	Ratio of indirect to direct effect
Average Trading Volume	7.81	5.77×10^−15^	3.31%	0.03
cross-sectional standard deviation in trading volume	6.11	1×10^−9^	4.22%	0.04
number of positive changes	7.24	4.41×10^−13^	14.89%	0.18
number of negative changes	13.51	0	28.73%	0.40

To empirically validate our full set of hypotheses, we run panel logistic regressions, which use within-sector variation across time and cross-sectional variation to adjust the standard errors. We considered both fixed effects models and between-effects models. Fixed effects models assume the unobserved variables differ between the sectors but are constant across time for the same sector [21]. This assumption is unrealistic because industry dynamics do change over time. Between effects models assume that the unobserved variables differ over time, but are constant across sectors for the same time period. Conceptually, this is as if each unobserved variable has a single mean value per time period. As a result, there is substantial loss of data variation. Because random effects models assume that unobserved variables may either remain constant or change over time, we run panel logistic regressions with random effects.

The independent variables are all lagged with one month. We present the results so that the readers first see clearly the curvilinear effects in a series of basic models.

In table 6, model 1 consists of the control variables as well as the density measures (i.e. number of common stocks). Our focus is on each sector experiencing entry. The key control variables remain statistically significant throughout. A sector is more likely to experienced IPO entry if there are IPO entries in the previous period. There is strong statistical support for the notion that entry rate is affected by cognitive legitimacy and competition as documented by Haveman [16]. Comparing model 2 to 5 with model 1, we see that there are evidence that the trading volume variables partially mediates the relationship between the density measures and IPO entry rate.

**Table 6:** Panel Logistic regressions (Basic models).

	Model_1	Model_2	Model_3	Model_4	Model_5
Constant	−2.44	−2.84	−3.35	−2.67	−3.14
	(17.62)***	(18.53)***	(19.47)***	(17.29)***	(18.88)***
Lag of number of IPOs	0.72	0.67	0.65	0.71	0.69
	(17.25)***	(15.82)***	(15.38)***	(16.94)***	(16.41)***
Lag of number of common stocks	5.48×10^−3^	4.93×10^−3^	5.43×10^−3^	5.06×10^−3^	5.42×10^−3^
	(5.69)***	(4.90)***	(5.27)***	(4.96)***	(5.21)***
Lag of number of common stocks squared	−8.26×10^−6^	−7.39×10^−6^	−7.74×10^−6^	−7.72×10^−6^	−7.89×10^−6^
	(5.04)***	(4.39)***	(4.57)***	(4.54)***	(4.61)***
Lag of average monthly stock return	1.09	1.1	1.05	1.12	1.13
	(3.24)***	(3.24)***	(3.11)***	(3.30)***	(3.31)***
Lag of cross-sectional standard deviation of stock returns	0.18	−0.13	−0.18	0.02	−0.18
	−0.45	−0.32	−0.43	−0.05	−0.45
Lag of average monthly trading volume		1.67	4.79		
		(10.43)***	(10.52)***		
Lag of average monthly trading volume squared			−3.45		
			(7.17)***		
Lag of cross-sectional standard deviation of trading volume				0.97	4.6
				(6.42)***	(10.60)***
Lag of cross-sectional standard deviation of trading volume squared					−4.03
					(8.85)***
Observations	18374	18249	18249	18249	18249
Number of sic_2	76	76	76	76	76

Absolute value of z statistics in parentheses,* significant at 10%; ** significant at 5%; *** significant at 1%.

In model 6 and 7 in Table 7, we show that there is a statistically significant positive correlation between IPO entry rate and the lag of number for positive changes (p value <0.01). In model 8 and 9, the correlation between the IPO entry rate and the square term of the positive changes is significantly negative (p value<0.01). In model 10 and 11 in Table 7, we show that there is a statistically significant positive correlation between IPO entry rate and the lag of number for negative changes (p value <0.01). In model 12 and 13, the correlation between the IPO entry rate and the square term of the negative changes is significantly negative (p value<0.01). The fact that the coefficients are significantly positive, for both model 6 and 7 as well as model 10 and 11, indicates that the amount of changes has a positive impact on IPO entry rate irrespective of whether the change is positive or negative. Since the coefficient of the positive changes is larger than the negative changes, the “good news” still has a higher positive impact on IPO entry rate than the “bad news”. However, the correlation between the IPO entry rate and the square term of the changes (both negative and positive) are both significantly negative (p value<0.01), which means that there is a curvilinear relationship between the changes (positive and negative changes) and IPO entry rate. The shape of this curvilinear relationship implies that when the amount of changes is small, the market will be encouraged by increasing the amount of changes and the IPO entry rate increases. However, after the amount of changes reach a certain point, the market will suffer from the increased amount of changes and the IPO entry rate will drop.

**Table 7:** Panel Logistic regressions (Extended models).

	Model_6	Model_7	Model_8	Model_9	Model_10	Model_11	Model_12	Model_13
Constant	−3.93	−3.61	−4.31	−3.99	−3.83	−3.54	−4.08	−3.8
	(27.17)***	(27.21)***	(32.82)***	(33.50)***	(26.15)***	(26.29)***	(30.50)***	(31.01)***
Lag of number of IPOs	0.64	0.69	0.62	0.66	0.63	0.67	0.64	0.68
	(15.33)***	(16.41)***	(14.84)***	(15.92)***	(15.00)***	(16.08)***	(15.16)***	(16.20)***
Lag of number of common stocks	3.58×10^−3^	3.54×10^−3^	1.86×10^−3^	1.82×10^−3^	3.73×10^−3^	3.71×10^−3^	2.07×10^−3^	1.96×10^−3^
	(3.78)***	(3.72)***	(1.92)*	(1.86)*	(3.91)***	(3.88)***	(2.10)**	(1.97)**
Lag of number of common stocks squared	−6.29×10^−6^	−6.34×10^−6^	−3.56×10^−6^	−3.51×10^−6^	−6.52×10^−6^	−6.58×10^−6^	−3.93×10^−6^	−3.8×10^−6^
	(3.95)***	(3.96)***	(2.19)**	(2.14)**	(4.09)***	(4.12)***	(2.36)**	(2.27)**
Lag of average monthly stock return	0.39	0.53	−0.02	0.12	1.26	1.33	1.41	1.47
	−1.06	−1.43	−0.06	−0.31	(3.81)***	(3.96)***	(4.26)***	(4.41)***
Lag of cross-sectional standard deviation of stock returns	0.32	0.26	0.58	0.52	0.14	0.12	0.2	0.18
	−0.76	−0.63	−1.38	−1.23	−0.35	−0.29	−0.51	−0.45
Lag of average monthly trading volume	4.81		4.48		4.61		4.31	
	(10.52)***		(9.56)***		(10.07)***		(9.24)***	
Lag of average monthly trading volume squared	−3.4		−3.14		−3.27		−3.06	
	(7.07)***		(6.47)***		(6.77)***		(6.29)***	
Lag of cross-sectional standard deviation of trading volume		4.43		4.03		4.26		3.9
		(10.19)***		(9.04)***		(9.71)***		(8.72)***
Lag of cross-sectional standard deviation of trading volume squared		−3.77		−3.5		−3.71		−3.44
		(8.28)***		(7.62)***		(8.11)***		(7.46)***
Lag of number of positive changes	1.68	1.52	6.43	6.38				
	(8.60)***	(7.72)***	(11.16)***	(11.02)***				
Lag of number of positive changes squared			−4.77	−4.85				
			(8.92)***	(9.00)***				
Lag of number of negative changes					1.49	1.4	4.67	4.71
					(8.02)***	(7.51)***	(8.55)***	(8.64)***
Lag of number of negative changes squared							−3.07	−3.19
							(6.16)***	(6.42)***
Observations	18249	18249	18249	18249	18249	18249	18249	18249
Number of sic_2	76	76	76	76	76	76	76	76

Absolute value of z statistics in parentheses, * significant at 10%; ** significant at 5%; *** significant at 1%.

## Conclusion

In this research, we find that having too many comparable stocks may not be a good thing in one aspect of the stock market: IPO entry rate. This finding is consistent with prior research in economic sociology that suggests the financial markets are boundedly rational and that the stock markets (or the market participants collectively) economize on information processing. Drawing on sociological theories [3], [16], we document that density generates information that affects the IPO entry rate and that too much information can also discourage entry because the stock markets is boundedly rational. Thus, there is a curvilinear effect that is independent of competition and legitimacy. Our results, using multiple proxies for information, are robust. We constructed our proxy variables for information based on trading data arising from supply-demand transactions including trading volume, changes in stock returns and the number of comparable stocks. Our choice of proxy variables is supported by recent research that suggests that processed information comes more from transactions than from external news [22].

## Supporting Information

Footnotes S1 (PDF)

Table S1 IPO distribution over time.
(PDF)
