# Supplementary material for: Comparable Stocks, Boundedly Rational Stock Markets and IPO Entry Rates
Source: PLoS One. 2013 May 17;8(5):e61474. doi: 10.1371/journal.pone.0061474 (PMC3656873; doi:10.1371/journal.pone.0061474)
Supplement: Footnotes S1 — (PDF) [file pone.0061474.s001.pdf]

# Footnotes S1

[1] For related work on sociological theorizing and IPOs, see Chok[1,2,3].

[2] There are exceptions. Fama and French [4] argue that the costs of equity affect firms' abilities to list on the stock exchange. Pastor and Veronesi [5] argue similarly that the number of initial public offerings is related to stock market conditions. Finally, Helwege and Liang [6] suggest that investor optimism affects the number of initial public offerings.

[3] This is in contrast to Pollock and Rindova [7],who argue that information provided by information intermediaries legitimizes firms going public. But their results are inconclusive. In fact, their results suggest that the proportion of positive information didn't really matter. Rather what matters is simply that there is information available. Likewise, Johnson, Ellstrand, Dalton & Dalton [8] found that financial press' rating positively influences stockholder's wealth. This relationship holds true for both favorable and unfavorable ratings.

[4] We use partial correlations as a diagnostic tool to make sure that the correlations between variables are not spurious. Spurious correlations can happen when, for example, variable z's correlation with variable y disappears when variable z enters into the equation. By controlling for the presence of a set of control variables, partial correlation help identify the true relationship between two variables after accounting for controls. The diagnostic checks provide assurances that correlations among our selected variables are neither excessive nor spurious. For references, refer to prior work authored by Dror and colleagues [9,10,11,12,13]

## Reference


1. Chok JI, Sun Q (2007) Determinants of idiosyncratic volatility for biotech IPO firms. Financial Management 36: 107-122.
2. Chok JI (2009) Regulatory dependence and scientific advisory boards. Research Policy 38: 710-725.
3. Chok JI (2009) Raising capital with uncertainty. Science and Innovation Policy, 2009 Atlanta Conference. pp. 1-6.
4. Fama EF, French KR (2004) New lists: Fundamentals and survival rates. Journal of Financial Economics 73: 229-269.
5. Helwege J, Liang N (2004) Initial public offerings in hot and cold markets. Journal of Financial and Quantitative Analysis 39: 541-569.
6. Pastor L, Veronesi P (2005) Rational IPO waves. Journal of Finance 60: 1713-1757.
7. Pollock TG, Rindova V (2003) Media legitimation effects in the market for initial public offerings. Academy of Management Journal 46: 631-642.
8. Johnson JL, Ellstrand AE, Dalton DR, Dalton CM (2005) The influence of the financial press on stockholder wealth: The case of corporate governance. Strategic Management Journal 26: 461-471.
9. Kenett DY, Shapira Y, Madi A, Bransburg-Zabary S, Gur-Gershgoren G, et al. (2011) Index Cohesive Force Analysis Reveals That the US Market Became Prone to Systemic Collapses Since 2002. PLoS ONE 6(4): e19378. doi:10.1371/journal.pone.0019378
10. Kenett DY, Tumminello M, Madi A, Gur-Gershgoren G, Mantegna RN, et al. (2010) Dominating Clasp of the Financial Sector Revealed by Partial Correlation Analysis of the Stock Market. PLoS ONE 5(12): e15032. doi:10.1371/journal.pone.0015032
11. Shapira Y, Kenett DY, Ben-Jacob E (2009) The index cohesive effect on stock market correlations. The European Physical Journal B-Condensed Matter and Complex Systems 72: 657-669.
12. Kenett DY, Preis T, Gur-Gershgoren G, Ben-Jacob E (2012) DEPENDENCY NETWORK AND NODE INFLUENCE: APPLICATION TO THE STUDY OF FINANCIAL MARKETS. International Journal of Bifurcation and Chaos 22.
13. Kenett DY, Raddant M, Zatlavi L, Lux T, Ben-Jacob E (2012) Correlations and Dependencies in the Global Financial Village. International Journal of Modern Physics: Conference Series: World Scientific. pp. 13-28.
