# Supplementary material for: Comparable Stocks, Boundedly Rational Stock Markets and IPO Entry Rates
Source: PLoS One. 2013 May 17;8(5):e61474. doi: 10.1371/journal.pone.0061474 (PMC3656873; doi:10.1371/journal.pone.0061474)
Supplement: Table S1 — IPO distribution over time. (PDF) [file pone.0061474.s002.pdf]

## Table S1 – IPO distribution over time

| Year | Number_IPO_firms |
|---|---|
| 1982 | 141 |
| 1982 | 141 |
| 1983 | 564 |
| 1984 | 737 |
| 1985 | 792 |
| 1986 | 808 |
| 1987 | 806 |
| 1988 | 812 |
| 1989 | 812 |
| 1990 | 813 |
| 1991 | 801 |
| 1992 | 794 |
| 1993 | 794 |
| 1994 | 809 |
| 1995 | 808 |
| 1996 | 809 |
| 1997 | 825 |
| 1998 | 835 |
| 1999 | 833 |
| 2000 | 848 |
| 2001 | 845 |
| 2002 | 830 |
| 2003 | 810 |
| 2004 | 796 |
| 2005 | 816 |
